# Characterization of Vegard strain related to exceptionally fast Cu-chemical diffusion in Cu$_2$Mo$_6$S$_8$ by an advanced electrochemical strain microscopy method

**DOI:** 10.1038/s41598-021-96602-2

**Published:** 2021-09-13

**Authors:** Sebastian Badur, Diemo Renz, Marvin Cronau, Thomas Göddenhenrich, Dirk Dietzel, Bernhard Roling, André Schirmeisen

**Affiliations:** 1 Institute of Applied Physics, Justus-Liebig-Universität Giessen, 35392 Giessen, Germany; 2 Department of Chemistry, Philipps-Universität Marburg, Hans-Meerwein-Straße 4, 35032 Marburg, Germany; 3 Center for Materials Research, Justus-Liebig-Universität Giessen, 35392 Giessen, Germany

**Keywords:** Batteries, Imaging techniques, Techniques and instrumentation

## Abstract

Electrochemical strain microscopy (ESM) has been developed with the aim of measuring Vegard strains in mixed ionic-electronic conductors (MIECs), such as electrode materials for Li-ion batteries, caused by local changes in the chemical composition. In this technique, a voltage-biased AFM tip is used in contact resonance mode. However, extracting quantitative strain information from ESM experiments is highly challenging due to the complexity of the signal generation process. In particular, electrostatic interactions between tip and sample contribute significantly to the measured ESM signals, and the separation of Vegard strain-induced signal contributions from electrostatically induced signal contributions is by no means a trivial task. Recently, we have published a compensation method for eliminating frequency-independent electrostatic contributions in ESM measurements. Here, we demonstrate the potential of this method for detecting Vegard strain in MIECs by choosing Cu$_2$Mo$_6$S$_8$ as a model-type MIEC with an exceptionally high Cu chemical diffusion coefficient. Even for this material, Vegard strains are only measurable around and above room-temperature and with proper elimination of electrostatics. The analyis of the measured Vegards strains gives strong indication that due to a high charge transfer resistance at the tip/interface, the local Cu concentration variations are much smaller than predicted by the local Nernst equation. This suggests that charge transfer resistances have to be analyzed in more detail in future ESM studies.

## Introduction

The growing efforts towards ecological sustainability put an ever increasing focus on the problem of energy storage, which is e.g. crucial to replace fossil fuels by renewable energies in the automotive sector. In this context, the optimization of electrochemical systems for energy storage does not only involve the application of materials with optimized chemical compositions^1^, but recent analysis also has highlighted the potential impact of the materials nanostructures, with e.g. grain boundaries or interface regimes showing improved transport properties^2–9^. Gaining nanoscale insight into the correlation between structure and electrochemical transport properties is thus not only a problem of fundamental interest^10–12^, but it is also considered essential for further improvement of high-performance energy storage devices^13^. Consequently, the conventional approaches to measure macroscopic transport parameters need to be complemented by analyses at the nanoscale.

In order to monitor electrochemical processes with nanoscale resolution, a number of experimental techniques have been developed in recent years^14–16^. In particular, atomic force microscopy (AFM) based methods are often used, since applying voltages to a conductive AFM tip allows to transfer classical concepts to the nanoscale, with the AFM tip acting as a nano-electrode. For example, in time-domain electrostatic force spectroscopy (TDESF), the time-dependent change of the eletrostatic field strength between an oscillating AFM-tip and the sample after voltage-switching can be correlated to the ion dynamics in solid electrolytes^17–20^. Alternatively, local ionic transport properties can be correlated to the growth characteristics of metallic nanoparticles, that develop by redox reactions under application of cathodic overpotentials between tip and sample^21–27^.

**Figure 1:**
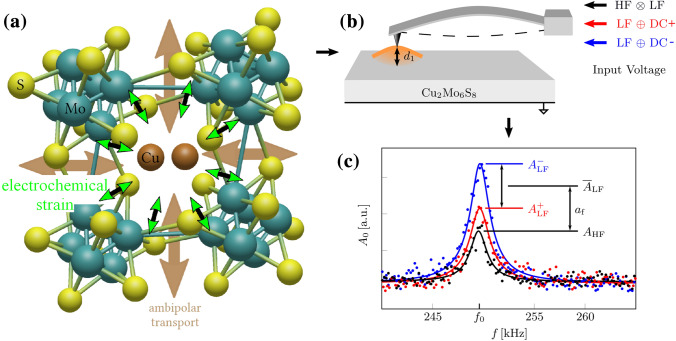
Scheme of material and setup. (**a**) Voltage driven ambipolar transport (indicated by larger brown arrows) on the Chevrel-phase Cu$_2$Mo$_6$S$_8$ induces Vegard-strain (indicated by small green arrows) due to change of Cu concentration (This subfigure was created using chemtool (1.6.14-4, http://ruby.chemie.uni-freiburg.de/ martin/chemtool/) and inkscape (1.0.2-6, https://inkscape.org)). (**b**) The input voltages and the Vegard-strain $d_1$ drive the cantilever oscillation. As external voltages we applied an LF modulated high frequency voltage (HF$\otimes$LF), a low frequency excitation with positive DC voltage offset (LF$\oplus$DC$^+$), and a low frequency excitation with negative DC voltage offset (LF$\oplus$DC$^-$). (**c**) Exemplary resonance curves measured at $300\,$K. A frequency related parameter $a_\text {f}$ can then be extracted from the resonance oscillation amplitudes $A_\text {LF}^+$, $A_\text {LF}^-$, and $A_{\text {HF}}$, that are resolved by band excitation and correspond to the different excitation voltages (see main text for details).

In the case of mixed ionic-electronic conductors (MIECs), acting as electrode materials in batteries, electrochemically induced changes in the chemical composition lead to Vegard strains, i.e. to the expansion or contraction of the material. For the local detection of such Vegard strains, the so-called electrochemical strain microscopy (ESM) technique has been developed^28,29^. In this technique, an ac voltage bias applied to the tip at the contact resonance frequency leads to local chemical composition changes underneath the tip and thus to a local Vegard strain (see Fig. 1). The oscillating Vegard strain drives the contact resonance oscillation of the tip. Consequently, the method makes use of a contact-resonance amplification of the signal^30–35^.

However, not only Vegard strains contribute to the measured ESM signals, but also electrostatic interactions between tip and sample^13,36,37^. In several cases, the electrostatically induced ESM signals were considered to be even the dominant signals^38,39^, and were, for instance, recently exploited for the analysis of local chemical distributions in solid state electrolytes^40^.

Furthermore, it has been shown that also local contact mechanics at the tip/sample interface and the resulting dynamic lever shape during the contact resonance oscillation exert a strong influence on the measured signals^36,41,42^.

Advanced methods for eliminating electrostatic effects are known from the closely related piezoresponse force microscopy (PFM): The so-called contact Kelvin Probe Force Microscopy (cKPFM) allows the quantitative measurement of electrostatic tip-sample interactions and its separation from electromechanical forces by continuous tracking of the local contact potential in combination with PFM measurements^39,43–45^. Another recently introduced approach relies on the utilization of a laser Doppler vibrometer (LDV)^38,46^. With this technique, non-local electrostatic signal contributions are effectively eliminated. Collins et al. showed that ESM hysteresis during voltage spectroscopy on ion conducting glasses, which is measured by conventional ESM/PFM setups and which had often been misunderstood as Vegard strain, can in fact be attributed to charge injection and electrostatic interactions^38^. However, to our knowledge, none of these advanced techniques have been successfully applied to mixed conducting electrode materials in order to probe Vegard strains.

In order to eliminate the influence of electrostatics and contact mechanical signal contributions without loosing resonance enhancement, we recently proposed a new compensation method based on band excitation, which exploits the distinct frequency dependences of Vegard strain and electrostatic contributions^47^. In principle, this approach is similar to recent works^33,48^, where Lock-in detection of the frequency-dependent ESM-signal in the low frequency range was discussed with respect to separation between Vegard strain and electrostatic interactions. In the case of the compensation method^47^ however, only two frequencies are used, where information on the Vegard strain is only contained in the low frequency signal, whereas the high-frequency signal is used as a reference to eliminate electrostatic contributions.

Here, we demonstrate the potential of this method for detecting Vegard strains in MIECs by choosing Cu$_2$Mo$_6$S$_8$ as a model-type MIEC material with an exceptionally high Cu chemical diffusion coefficient of $D_{Cu} = 3 \cdot 10^{-6}$ cm$^2$/s at 300K (compare Fig. S1). Note that this value is about 5 to 6 orders of magnitude higher than the lithium diffusion coefficient in the common LIB electrode material LiCoO$_2$ ($D_{Li} \approx 10^{-11} \, \text {to} \,10^{-12}$ cm$^2$/s)^49–51^. Thus, it is expected that the Vegard strains in Cu$_2$Mo$_6$S$_8$ at typical contact resonance frequencies of several 100 kHz are considerably higher than those in typical LIB electrode materials.

Cu$_2$Mo$_6$S$_8$ belongs to the family of Chevrel phases with the general formula M$_x$Mo$_6$T$_8$ (M = metal, T = S, Se), which are especially known for their remarkable intercalation chemistry^52–54^. Mo$_{6}$S$_8$ and Mo$_6$Se$_8$ do not only allow ultrafast and reversible insertion of monovalent cations like Li$^+$ or Na$^+$ but also of divalent cations like Cu$^{2+}$, Zn$^{2+}$ or Mg$^{2+}$^54–56^. The Chevrel phase family contains various promising candidates for the development of high-performance rechargeable batteries, which might serve as an alternative to lithium ion batteries (LIBs)^52,57–60^. Especially, Mg$_x$Mo$_6$S$_8$-based systems are of great interest due to the high theoretical capacity and the great disposability of magnesium^58^.

By applying the new compensation method^47^ to Cu$_2$Mo$_6$S$_8$, we demonstrate that a proper elimination of electrostatic contributions is essential for quantitative measurements of Vegard strains. Even with such a proper elimination, Vegard strains can only be detected around and above room temperature. This finding is related to charge transfer resistances at the interfaces between tip and MIECs, which are anticipated to strongly influence the results of electromechanical AFM-experiments^48^. In particular, our results give strong indication that a high charge transfer resistance at the tip/sample interface results in Cu concentration variations, which are much smaller than expected from the local Nernst equation.

## Results and discussion

All ESM measurements were performed on Cu$_2$Mo$_6$S$_8$ using an atomic force microscope under ultrahigh vacuum conditions, where the sample temperature *T* was systematically varied between $200\,$K and $400\,$K (see “Materials and methods” for details). As shown in Fig. 1b, all electrical voltages were applied to the conductive diamond tip of the cantilever, with the counter electrode at the backside of the sample set to ground potential. If, for instance, a negative voltage is applied to the tip, Vegard strain can build up in a process where the movement of Cu$^{2+}$ ions to the tip/sample-interface is coupled to the injection of electrons into the sample^61^.

The compensation method used in this work relies on applying different external voltages between the cantilever and the back-electrode of the sample. Detecting the peak values of the cantilever oscillations for each excitation basically allows to distinguish the different nanoscale contributions to the electromechanical response. More specifically, to quantify Vegard strain, three different excitations are applied. First, an amplitude modulated high frequency excitation is used as a reference, which exclusively contains signal contributions related to electrostatic forces acting on the cantilever since ambipolar diffusion is to slow to follow the high frequencies. In addition, also low frequency voltages with either positive or negative DC-bias are applied to the cantilever. In this case, surface displacements related to ambipolar diffusion can contribute to the signals, and as we will discuss later on, information about the Vegard strain can be gained from subtracting their average peak amplitudes from the reference amplitude, which yields the value $a_f$ as shown in Fig. 1. In addition, this approach can also be applied to eliminate signal contributions related to local contact mechanics and topography-crosstalk. In this case the tip sample contact needs to be described based on a Kelvin-Voigt model as detailed in^47^.

Figure 1c further illustrates how the three different excitation voltages are used in our experiments: (i) low frequency excitation with positive DC voltage offset (LF$\oplus$DC$^+$), (ii) low frequency excitation with negative DC voltage offset (LF$\oplus$DC$^-$), and (iii) an LF modulated high frequency voltage (HF$\otimes$LF). For each of these excitations, the corresponding resonant oscillation amplitudes $A_\text {LF}^+$, $A_\text {LF}^-$, and $A_{\text {HF}}$ (Fig. 1c) were resolved by band excitation (BE)^62,63^ and recorded in addition to the topography signal of the AFM.

For estimating the magnitude of the Vegard strain, compositional variations induced by the voltage-biased tip have to be integrated over the diffusion length of the experiment. Therefore, the Vegard strain is expected to be proportional to the diffusion length $L_D$^30^,

1 $$\begin{aligned} d_1(f,T)\propto L_D = \sqrt{\frac{D(T)}{2\pi f}} \end{aligned}$$

as long as $L_D$, which is dependent on both the temperature *T* and the excitation frequency *f*, is smaller than the tip diameter. At low frequencies ($f_\text {LF}\approx 250\,$kHz) and at high frequencies ($f_\text {HF}\approx 10\,$MHz), we obtain diffusion lengths of $L_D \approx 14 \mathrm{nm}$ and $L_D \approx 2 \mathrm{nm}$, respectively, Consequently, we expect significant contributions of Vegard strain to the ESM signal only at low frequencies, while the high-frequency measurements are dominated by electrostatic effects^47^.

Based on these considerations, the Vegard strain at 250 kHz is given to a very good approximation by $d_1 \approx a_\text {f}={\overline{A}}_\text {LF}-A_\text {HF}$, using the average LF amplitude ${\overline{A}}_\text {LF}=(A_\text {LF}^-+A_\text {LF}^+)/2$ and the reference amplitude $A_\text {HF}$, which is independent of both applied DC-voltages.

**Figure 2:**
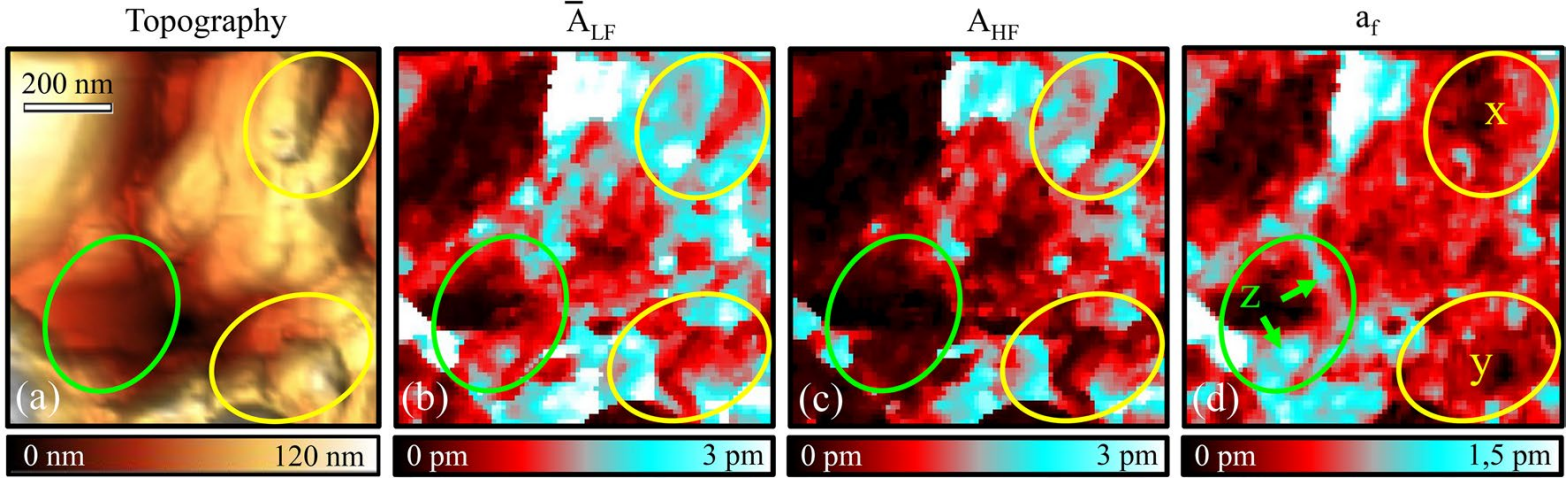
Electrochemical strain microscopy (ESM) on Cu$_2$Mo$_6$S$_8$ performed at room temperature with voltages of $U_\text {AC}=1\,$V and $U_\text {DC}=\pm 1\,$V. (**a**) Topography, (**b**) average low frequency response ${\overline{A}}_\text {LF}$, (**c**) high frequency response $A_\text {HF}$, and (**d**) the frequency related parameter $a_\text {f}$. Three regions have been marked to illustrate different strain related contrast (see main text for details).

To illustrate the compensation method, Fig. 2 shows spatially resolved measurements of ${\overline{A}}_\text {LF}$, $A_\text {HF}$, and $a_\text {f}$ in comparison with the topography. The ESM data was recorded for an $(0.8\,\mu \text {m}\times 0.8\,\mu \text {m})$ area, while voltages of $U_\text {ac}=1\,$V and $U_\text {dc}=\pm 1\,$V were applied. All resonance amplitudes have been derived by band excitation covering a frequency range from $80\,$kHz to $320\,$kHz with 20 times averaging.

From Fig.  2 we can see that the signals related to the cantilever oscillations are highly dependent on location: (x, y) In these two regions, we find that both ${\overline{A}}_\text {LF}$ and $A_\text {HF}$ show high and low oscillation amplitudes, while the corresponding signal amplitudes of $a_\text {f}$ remain relatively constant around 0.5 pm. This suggests that unwanted signal contributions related to electrostatics and topographic effects dominate both ${\overline{A}}_\text {LF}$ and $A_\text {HF}$, but are effectively eliminated in $a_\text {f}$, although both regions (x) and (y) show a certain roughness related to the very granular structure of the material. Thus we benefit from using a method that is less susceptible to mechanical cross-talk^47^. Finally, it is known that ambipolar diffusion depends on crystallographic orientation and grain size and can be favored at defects such as grain boundaries^64,65^. Such effects appear to be evident in region (z), where large values of ${\overline{A}}_\text {LF}$ and $a_\text {f}$, but not of $A_\text {HF}$, are observed, suggesting large contributions of Vegard strain to the low-frequency signals.

**Figure 3:**
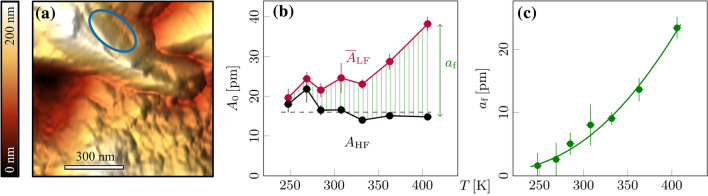
(**a**) Topography of the Cu$_2$Mo$_6$S$_8$ surface with an indication of the area under investigation in (**b**,**c**). (**b**) Linear plot of amplitudes $A_\text {HF}$ (black) and ${\overline{A}}_\text {LF}$ (red) averaged over the outlined region. The HF amplitude is constant over the temperature range, while the average LF amplitude increases with temperature. (**c**) The parameter $a_\text {f}$ extracted from (**b**) increases exponentially with temperature.

As a next step, we performed temperature-dependent ESM measurements in a range from $250\,$K up to $400\,$K on a homogeneous area on top of a single large grain, as indicated by the outline in Fig. 3. For a clear detection of signals also at low temperatures, we used $U_\text {ac}=2.5\,$V and $U_\text {dc}=\pm 2.5\,$V. We find that the average values of ${\overline{A}}_\text {LF}$ within the marked area are increasing with temperature, while $A_\text {HF}$ remains almost constant with an amplitude of about $16\,$pm (Fig. 3b). The small temperature dependence of $A_\text {HF}$ supports our argument given above that the $A_\text {HF}$ signal is governed by electrostatic interactions. For technical reasons (see “Materials and Methods” section), slight variations in $A_\text {HF}$ may result from differences in sensitivity and normal force. However, since both $A_\text {HF}$ and ${\overline{A}}_\text {LF}$ are equally affected by this, the effects largely cancel out when calculating $a_\text {f}$. Ultimately, $a_\text {f}$ increases strongly with increasing temperature and exhibits values ranging from roughly $2\,$pm at $250\,$K up to $25\,$pm at $400\,$K (Fig. 3c).

**Figure 4:**
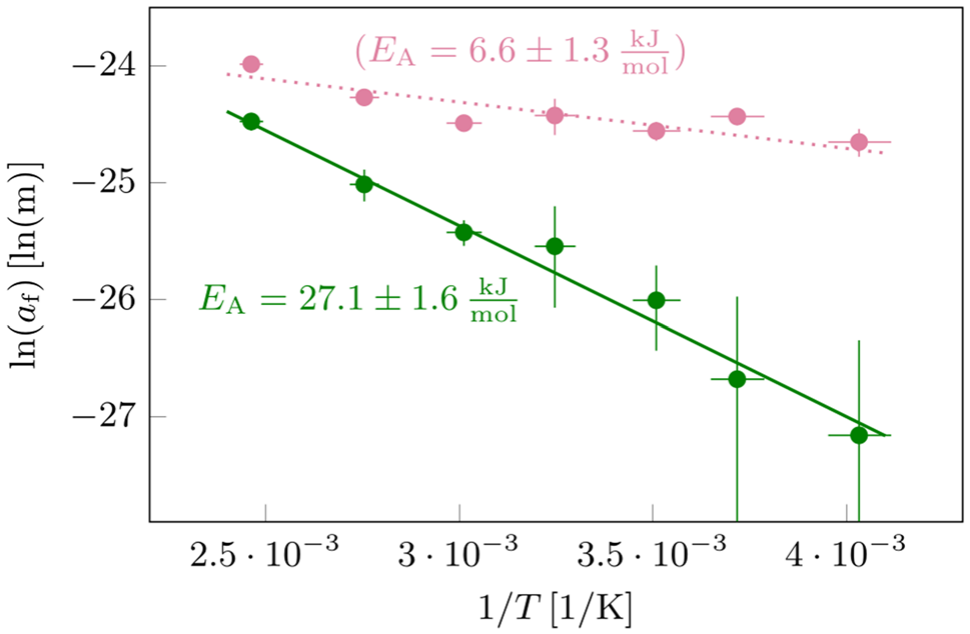
Arrhenius Plots of the ESM data recorded for a single grain (see Fig. 3): (Green) Frequency-dependent Vegard strain parameter $a_\text {f}$ plotted vs. the inverse temperature. (Red) Average LF amplitude ${\overline{A}}_\text {LF}$ plotted vs. the inverse temperature. In both cases, the slope represents an activation energy barrier $E_\text {A}$ for ambipolar diffusion with quantitative values as indicated in the figure. Please note that the temperature-dependent analysis of ${\overline{A}}_\text {LF}$ does not result in a meaningful energy barrier for ambipolar diffusion, but was rather added to highlight the problems, that can arise from temperature independent contributions, which significantly reduce the slope.

According to Eq. 1, the temperature dependence of the Vegard strain $a_\text {f}$ should be influenced by the temperature dependence of the chemical diffusion coefficient *D*(*T*). Macroscopic measurements of *D*(*T*) by means of impedance spectroscopy yield an Arrhenius-type temperature dependence $D(T)\propto e^{-E_\text {A}/\text {RT}}$ with an activation energy $E_\text {macro}=23.0\pm 1.0\,\frac{\text {kJ}}{\mathrm {mol}}$, (see Fig. S1 and related information in the Supporting Information). If the temperature dependence of the Vegard strain $a_\text {f}$ was exclusively due to temperature dependence of the chemical diffusion coefficient, the activation energy in the ESM experiments would be given by:

2 $$\begin{aligned} E_\text {A}=-2R\frac{\partial \ln a_f(T)}{\partial (1/T)} \end{aligned}$$

In Fig. 4, we show an Arrhenius plot of $a_\text {f}(\text {T})$ (green symbols) yielding an activation energy of $E_\text {A}=27.1\pm 1.6\,\frac{\text {kJ}}{\mathrm {mol}}$, which is close to the macroscopic value for *D*(*T*). We added also the data for the low-frequency signal ${\overline{A}}_\text {LF}$ (red data points) to Fig. 4. The weak temperature dependence of ${\overline{A}}_\text {LF}$ as compared to $a_\text {f}(\text {T})$, as already evident in Fig. 3, points to the significant contribution of electrostatics to the ${\overline{A}}_\text {LF}$ and emphasizes the importance of the compensation method.

Next we analyze the results obtained for the Vegard strains $d_1 \approx a_f$ in relation to theoretical predictions. In Balke et al.^66^, the following relation between the Vegard strain $d_1$ and the applied ac voltage $U_{ac}$ is given:

3 $$\begin{aligned} d_1 = 2 (1+\nu ) \, \beta \, \frac{U_{ac}}{\eta }\sqrt{\frac{D(T)}{2\pi f}}\end{aligned}$$

Here, $\nu$ and $\beta$ denote Poisson’s ratio and the Vegard coefficient, respectively. The Vegard coefficient relates the relative expansion or contraction of the crystal lattice to the relative variation in the Cu concentration. The quantity $\eta = U_{ac} \cdot c_{Cu} / \Delta c_{Cu}$ relates the applied ac voltage to the periodic relative variation in the Cu concentration at the tip/sample interface. Consequently, the relative Cu concentration variation can be written as:

4 $$\begin{aligned} \frac{\Delta c_{Cu}}{c_{Cu}} = \frac{d_1}{2 (1+\nu ) \, \beta \, \sqrt{\frac{D(T)}{2\pi f}}} \end{aligned}$$

Thus, Eq. (3) and the eq. for $\eta$ imply that $V_{ac}$ leads to a periodic variation of $\Delta c_{Cu}/c_{Cu}$ and, in turn, to a periodic variation of the Vegard strain. Typical values for Poisson’s ratio are around 0.25. In order to estimate the Vegard coefficient $\beta$, we average over all crystal orientations of the hexagonal Cu$_2$Mo$_6$S$_8$ lattice by considering the total volume of the crystal lattice *V* as a function of the Cu content *x*. This approach is required since the radial chemical diffusion below the AFM-tip always leads to a geometrical averaging effect, even in a single measurement.

From x-ray diffraction data in Ref.^67^, the estimated Vegard coefficient around $x = 2$ is $\beta \approx \frac{1}{3} \frac{dlnV}{dlnx} = 0.03$. With $D_{Cu}(300~K) = 3 \cdot 10^{-6}\,$cm$^2$/s, $\omega \approx 2\pi \cdot 250 \mathrm{kHz}$ and $d_1(300 K) \approx a_f (300~$K) = 5 pm, we find that $\frac{\Delta c_{Cu}}{c_{Cu}}(300 K) =\frac{\Delta x_{Cu}}{x_{Cu}}(300 K)\approx$ 0.005.

From the galvanostatic titration curves shown in Ref.^67^, it is evident that such tiny concentration changes correspond to only a few mV changes in the thermodynamic reduction potential of Cu$_2$Mo$_6$S$_8$. Considering the applied ac voltage $U_{ac} = 2.5 V$, this implies that there must be a huge resistance $R_{CT}$ (and thus overpotential) for electronic charge transfer at the tip/sample interface. In the ESI Part B, the overall resistance $R_{total} = R_{CT} + R_D$, with $R_D$ denoting the spreading diffusion resistance of the probed subvolume, was estimated based on the assumption that the measured Vegard strain depends essentially on the charge flow and that orientation-dependent strain effects are of second order. As shown in the online supporting material (Part B), $R_{total}$ is around $5\cdot 10^{10}~\Omega$. For comparison, the spreading diffusion resistance of the probed subvolume below the tip was estimated to be around $3\cdot 10^{5}~\Omega$. i.e. about five orders of magnitude lower than $R_{CT}$ (see Supporting Material, Part B). This implies that $R_{CT} \approx R_{total} \approx 5\cdot 10^{10}~\Omega$. While the absolute charge transfer resistance is high, the area-normalized value $10^{-1}~\Omega cm^2$ is comparably low (nominal AFM tip radius of about 10 nm). These estimations confirm that the charge flow and Vegard strain are limited by interfacial charge transfer rather than chemical diffusion in the probed subvolume, which is in contrast to the assumption made in former ESM studies that charge transfer resistances were small or even negligible^66,68^ , but are in line with a recent analysis of charge transfer resistances in AFM experiments^48^. Thus, in future ESM measurements, more attention has to be paid to contact or charge transfer resistances at the tip/sample interface. If these resistances can be reduced, e.g. by using suitable tips and/or by removing possible resistive layers at the sample surface, larger ESM signals at lower ac voltages should be detectable.

## Conclusion

A recently introduced compensation method for the separation of electrostatic and electromechanical signal (e.g. Vegard strain) contributions in electrochemical strain microscopy (ESM) has been applied to the mixed ion/electron conductor (MIEC) Cu$_2$Mo$_6$S$_8$, a model-type material with an extraordinary high copper diffusion coefficient of $D_{Cu} = 3 \cdot 10^{-6}$ cm$^2$/s. To our knowledge, this work presents the first temperature-dependent Vegard strain measurements by an advanced ESM method. The potential of the new method was demonstrated by temperature-dependent measurements on a single grain, clearly achieving a successful separation of a temperature-independent electrostatic contribution and a thermally activated Vegard strain. We found that even for this material, the contributions of Vegard strain and of electrostatics to the overall ESM signal are in the same range at room temperature. Thus, elimination of eletrostatic contributions is crucial for detecting and analyzing Vegard strain contributions. Our results give strong indication that in the case of Cu$_2$Mo$_6$S$_8$, the Vegard strain is limited by a large charge transfer resistance at the tip/sample interface and not by chemical diffusion in the probed subvolume of the sample. Thus, in future ESM measurements, more attention should be paid to contact/charge transfer resistances at the tip/sample interface.

## Methods

All temperature-dependent AFM measurements have been performed under ultra-high vacuum conditions using a variable temperature atomic force microscope (VT-AFM) by ScientaOmicron. The temperature-dependent experiments have been performed as a series of spatially resolved ESM measurements at fixed temperatures between $200\,$K up to $400\,$K. During temperature changes, we have carefully monitored and tracked the sample position to eliminate drift effects. Thereby, the exact same area of the sample was analyzed at each temperature.

The AFM was operated in contact mode using a normal force setpoint of $10\,$nN. After each temperature change, the laser spot position on the cantilever was readjusted, a procedure with potentially minor influence on the normal force. Throughout the experiments we used commercial cantilevers with a conductive and non-abrasive single crystal diamond tip (Adama Innovations, AD-E-0.5-AS). These cantilevers are characterized by a nominal tip radius of $R\lesssim 10\,$ nm, a spring constant of $0.5\,$N/m, and a free resonance frequency of $30\,$kHz.

## Supplementary information

Supplementary Information.
