# Supplementary material for: Characterization of Vegard strain related to exceptionally fast Cu-chemical diffusion in Cu2Mo6S8 by an advanced electrochemical strain microscopy method
Source: Sci Rep. 2021 Sep 13;11:18133. doi: 10.1038/s41598-021-96602-2 (PMC8438055; doi:10.1038/s41598-021-96602-2)
Supplement: Supplementary file 1 — Supplementary Information. [file 41598_2021_96602_MOESM1_ESM.pdf]

Supporting Information: Characterization of Vegard Strain related to Exceptionally Fast Cu-Chemical Diffusion in $Cu_2Mo_6S_8$ by an Advanced Electrochemical Strain Microscopy Method

Sebastian Badur,[†] Diemo Renz,[‡] Marvin Cronau,[‡] Thomas Göddenhenrich,[†] Dirk Dietzel,[†,¶] Bernhard Roling,[*,‡] and André Schirmeisen[*,†,¶]

†*Institute of Applied Physics, Justus-Liebig-Universität Gießen, Heinrich-Buff-Ring 16, 35392 Gießen, Germany*

‡*Department of Chemistry, Philipps-Universität Marburg, Hans-Meerwein-Straße 4, 35032 Marburg, Germany*

¶*Center for Materials Research, Justus-Liebig-Universität, 35392 Gießen, Germany*

E-mail: roling@staff.uni-marburg.de; andre.schirmeisen@ap.physik.uni-giessen.de

Supporting Information A

Complementary macroscopic impedance measurements have been performed (Fig. S1), using a sandwich cell (i.e. a system structured as solid electrolyte - mixed conductor - solid electrolyte). In such a system, electrons are blocked by the copper ion solid electrolyte and a concentration profile inside the mixed conducting $Cu_2Mo_6S_8$ is built up, until a stationary

state is reached at very low frequencies. The ambipolar diffusion coefficient can then be obtained from the measured impedance data by fitting a model (Eq. S1) developed by Jamnik, Maier and Pejovnik.[1]

$$
\begin{aligned}
&Z(\omega) = \\
&R_{\text{elec}} + \left(\frac{1}{R_{\text{ion}} + R_{\text{eon}}}\right)^{-1} \\
&+ \frac{\left(R_{\text{ion}} - \left(\frac{1}{R_{\text{ion}}+R_{\text{eon}}}\right)^{-1}\right) \tanh \sqrt{\frac{i\omega L^2}{4D_{\text{Cu}}}}}{\sqrt{\frac{i\omega L^2}{4D_{\text{Cu}}}} + \frac{i\omega (R_{\text{ion}}+R_{\text{eon}}) C_{\text{el}}}{2} \tanh \sqrt{\frac{i\omega L^2}{4D_{\text{Cu}}}}}
\end{aligned}
\tag{S1}
$$

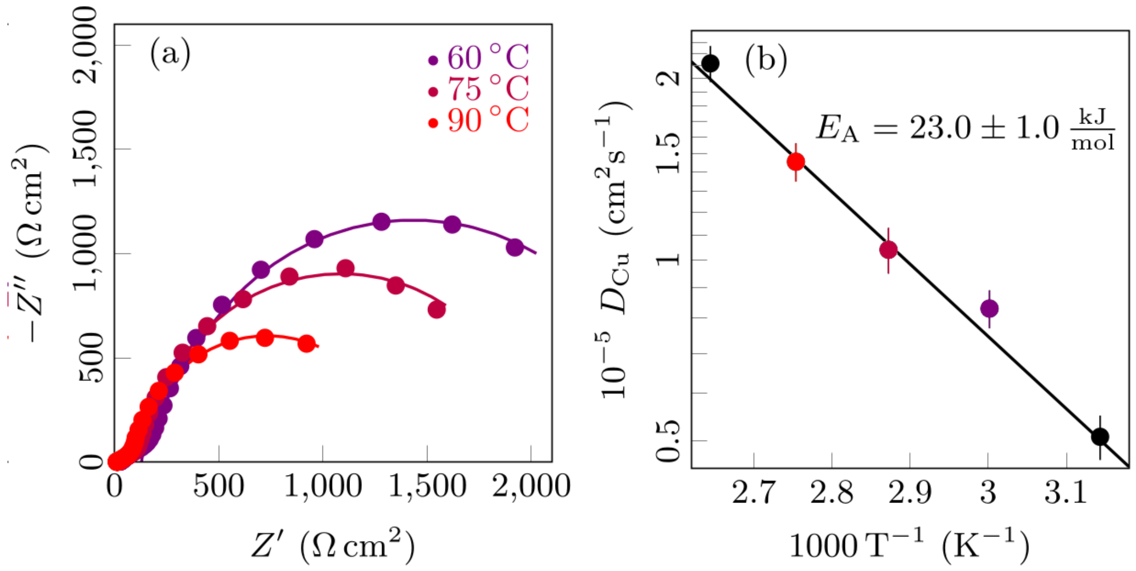

Figure S1: (a) Macroscopically measured impedance spectra for three different temperatures, fitted by Eq. S1. (b) The ambipolar diffusion coefficient follows an Arrhenius law with an activation energy of $23.0\,\frac{\text{kJ}}{\text{mol}}$. The diffusion coefficient of copper was extrapolated to 300 K, yielding $3 \cdot 10^{-6}\,\text{cm}^2/\text{s}$.

Here, Z is the overall impedance, ω is the angular frequency, R_{elec} is the electrolyte resistance, R_{ion}, and R_{eon} are the ionic and electronic resistance, respectively, of the mixed conductor, L is the thickness of the mixed conducting sample, D_{Cu} is the ambipolar diffusion coefficient of copper and C_{el} is the electrode capacitance. The temperature slope of the ambipolar diffusion coefficient then gives an activation energy of $E_{\text{macro}} = 23.0 \pm 1.0\,\frac{\text{kJ}}{\text{mol}}$ (Figure S1b). The diffusion coefficient of copper was extrapolated to 300 K, yielding $3 \cdot$

$10^{-6}\,\mathrm{cm^2/s}$.

Supporting Information B

We estimate the total resistance $R_{total} = R_{CT} + R_D$ during the ESM experiments:

$$R_{total} = |Z_{total}| = \frac{1}{C_{total}\omega} \tag{S2}$$

with the total capacitance

$$C_{total} = \frac{\Delta n \cdot 2F}{U_{ac}} \tag{S3}$$

Here, Z_{total} denotes the total complex impedance, while R_{CT} and R_D are the electronic charge transfer resistance at the tip/sample interface and the spreading diffusion resistance of the probed subvolume, respectively. Δn denotes the variation of the the molar amount of Cu in the probed subvolume, which can be written a:

$$\Delta n = c_{Cu} \cdot \frac{\Delta c_{Cu}}{c_{Cu}} \cdot V_P \tag{S4}$$

Here, c_{Cu} is the bulk copper concentration and V_P ist the probed volume. The bulk copper concentration is given by:

$$c_{Cu} = \frac{x_{Cu} \cdot N_U}{V_{EZ} \cdot N_A} = 12 \cdot 10^3\ \mathrm{mol/m^3} \tag{S5}$$

where $x_{Cu} = 2$ is the copper content, $N_U = 3$ is the number of formular units per unit cell and $V_{EZ} = 820$ Å^3 is the volume of one unit cell.[2] The probed volume is approximated by a semi-ellipsoid with:

$$V_P \approx 2/3 \cdot \pi \cdot r_{tip}^2 \cdot l_{eff} \tag{S6}$$

For the AFM tip radius, we use the nominal value $r_{tip} \approx 10nm$. According to Ref.[3] , the effective diffusion length l_{eff} in an ac micro-/nano-electrode experiment is given by

$$l_{eff} = 4 \cdot \sqrt{\frac{D_{Cu}}{\omega}} \cdot \sqrt{(\Phi_1)^2 + (\Phi_2)^2} \tag{S7}$$

where Φ_1 and Φ_2 are functions of the dimensionless frequency $(r_{tip}\omega/D)$. We use pre-calculated values from Ref[3] which are $\Phi_1 = 0.22$ and $\Phi_2 = 0.06$.

Combining Eqs. S2 to S7 and taking into account the relative Cu concentration variation at 300 K, $\frac{\Delta c_{Cu}}{c_{Cu}} \approx 0.005$, estimated from the Vegard strain, we obtain:

$$R_{total} \approx \frac{3U_{ac}}{2\pi r_{tip}^2 \cdot l_{eff} \cdot \Delta c_{Cu} \cdot zF\omega} = 5 \cdot 10^{10}\Omega \tag{S8}$$

Next, we compare this overall resistance to the spreading diffusion resistance R_D of the probed subvolume, which can be written as:[4]

$$\frac{R_D}{R_\infty} = \frac{|Z_D|}{R_\infty} = \left(\frac{r_{tip}}{l_{eff}} + 1\right)^{-1} \tag{S9}$$

with the steady state diffusion resistance

$$R_\infty = \frac{1}{4r_{tip}\sigma_d} \tag{S10}$$

and

$$\sigma_d = \frac{(2F)^2 \cdot c_{Cu} \cdot D_{Cu}}{RT} \tag{S11}$$

With $D_{Cu} = 3 \cdot 10^{-6}$ cm^2/s and $T = 300$ K, this results in:

$$R_D \approx 3 \cdot 10^5 \ \Omega \tag{S12}$$

Since the overall resistance R_{total} is more than five orders of magnitude larger than R_D,

it follows for the charge transfer resistance that $R_{CT} \approx R_{total}$.

Supporting Information C

$Cu_2Mo_6S_8$ was synthesized from its elements by heating in a sealed silica tube for a couple of days. After that, the material was quenched, ground, and cold-pressed in a procedure similar to Dudley et al.[5,6]

$Rb_4Cu_{16}I_7Cl_{13}$ was synthesized by stoichiometric reaction of RbCl (Chempur, 99.9 %), CuCl (Alfa Aesar, 99.999 %) and CuI (Acros Organics, 99.995 %). The synthetic steps were performed under argon atmosphere. The powders were mixed in an agate mortar and filled into a quartz ampoule. The ampoule was sealed under vacuum and heated to 750 °C with heating rate of 30 °C/h. The temperature was kept constant for 12 h until it was quenched to room temperature. The powder was pressed with 106 MPa using a P/O Weber precision press tool.

For the macroscopic AC impedance measurements a sandwich cell was assembled. To this end, the prepared $Cu_2Mo_6S_8$ powder was pressed into a 6 mm pellet by applying a pressure of 106 MPa at room temperature for 15 minutes, using a P/O Weber precision press tool with stainless steel discs. The thickness of the $Cu_2Mo_6S_8$ pellet was measured using a micrometer screw gauge from Mitutoyo to be 1.2 mm. Then, the $Cu_2Mo_6S_8$ was stacked between two prepressed $Rb_4Cu_{16}I_7Cl_{13}$ electrolyte[7] pellets and placed between two layers of copper foil. To ensure good contact between the layers, the sample was mounted into an air-tight two-electrode sample cell using a spring to exert pressure on the sample. A Novocontrol Alpha-AK impedance analyzer was used over a frequency range of 10^5 Hz to 10^{-4} Hz, using an AC voltage of 5 mV. The measurements were carried out at temperatures between 45 and 105 °C, controlled by a Novocontrol Quatro Cryosystem. Fitting of the impedance spectra was carried out by means of the RelaxIS software (RHD instruments), using a model developed by Jamnik, Maier and Pejovnik.[1]

References

(1) Jamnik, J.; Maier, J.; Pejovnik, S. A powerful electrical network model for the impedance of mixed conductors. *Electrochimica Acta* **1999**, *44*, 4139–4145.

(2) Fischer, C.; Gocke, E.; Stege, U.; Schoellhorn, R. Molybdenum Cluster Chalcogenides: In Situ X-Ray Studies on the Formation of CuxMo6S8 via Electron/Ion Transfer. *Journal of Solid State Chemistry* **1993**, *102*, 54–68.

(3) Fleischmann, M.; Pons, S. The behavior of microdisk and microring electrodes. Mass transport to the disk in the unsteady state. *Journal of Electroanalytical Chemistry* **1988**, *250*, 285–292.

(4) Rotenberg, Z. A.; Dribinskii, A. V.; Lukovtsev, V. P.; Khozyainova, N. S. Electrochemical impedance of microelectrodes. *Russian Journal of Electrochemistry* **2000**, *36*, 879–882.

(5) Dudley, G. J.; Cheung, K. Y.; Steele, B. C. H. Solid-state electrochemical studies of the mixed conductor CuxMo6S8- y. I. Partial copper ion conductivity and chemical diffusion. *Journal of Solid State Chemistry* **1980**, *32*, 259–267.

(6) Badur, S.; Renz, D.; Göddenhenrich, T.; Ebeling, D.; Roling, B.; Schirmeisen, A. Voltage- and Frequency-Based Separation of Nanoscale Electromechanical and Electrostatic Forces in Contact Resonance Force Microscopy: Implications for the Analysis of Battery Materials. *ACS Applied Nano Materials* **2020**,

(7) Takahashi, T.; Yamamoto, O.; Yamada, S.; Hayashi, S. Solid-State Ionics: High Copper Ion Conductivity of the System CuCl-CuI-RbCl. *Journal of the Electrochemical Society* **1979**, *126*, 1654.
